# Immunotherapy for cancer in the central nervous system: Current and future directions

**DOI:** 10.1080/2162402X.2015.1082027

**Published:** 2015-09-11

**Authors:** David C. Binder, Andrew A. Davis, Derek A. Wainwright

**Affiliations:** a Commitee on Cancer Biology; b Department of Pathology, The University of Chicago, Chicago, IL, USA; c Department of Medicine, Northwestern University Feinberg School of Medicine, Chicago, IL, USA; d Department of Neurological Surgery, Northwestern University Feinberg School of Medicine, Chicago, IL, USA

**Keywords:** Brain metastases, glioblastoma, glioma, IDO, T cell therapy, vaccination

## Abstract

Glioblastoma multiforme (GBM) is the most common primary brain tumor in adults and still remains incurable. Although immunotherapeutic vaccination against GBM has demonstrated immune-stimulating activity with some promising survival benefits, tumor relapse is common, highlighting the need for additional and/or combinatorial approaches. Recently, antibodies targeting immune checkpoints were demonstrated to generate impressive clinical responses against advanced melanoma and other malignancies, in addition to showing potential for enhancing vaccination and radiotherapy (RT). Here, we summarize the current knowledge of central nervous system (CNS) immunosuppression, evaluate past and current immunotherapeutic trials and discuss promising future immunotherapeutic directions to treat CNS-localized malignancies.

## Introduction

Glioma is the most common primary malignant brain tumor, accounting for nearly 80% of cases in adults. Glial-derived tumors are classified based on histologic subtype, which include glial fibrillary acidic protein positive (GFAP+) astrocytic tumors, oligodendrogliomas, ependymomas and a mixture of the subtypes.^1^ Of these, astrocytic glioma grade IV, otherwise referred to as GBM, is the most common and deadly subtype with a median survival of 14.6 mo post-diagnosis and an average 5-year survival rate of less than 5%.^2,3^ Current treatments that combine resection, RT and chemotherapy are unable to prevent tumor recurrence based on residual disease originating from the invading margins/inoperable surgical bed. Despite previous translational efforts that include new approaches for gene therapy, targeted chemotherapeutics and/or radiotherapeutic modalities, the standard of care for newly diagnosed GBM has remained unchanged for the past 10 y, highlighting the need for better treatment options. Also, there is no standard of care treatment for patients with recurrent GBM. The prevalence of metastatic tumors in the CNS greatly exceeds the number of GBM cases, yet, overall survival (OS) is similarly dismal. In this review, we discuss historical efforts, as well as new and/or expanded approaches that include vaccination, immune checkpoint blockade, adoptive T cell transfer, as well as combinatorial immunotherapy for the rationale design to durably control aggressive tumors in the CNS.

## CNS tumors and Immunosuppression

**Figure 1.**
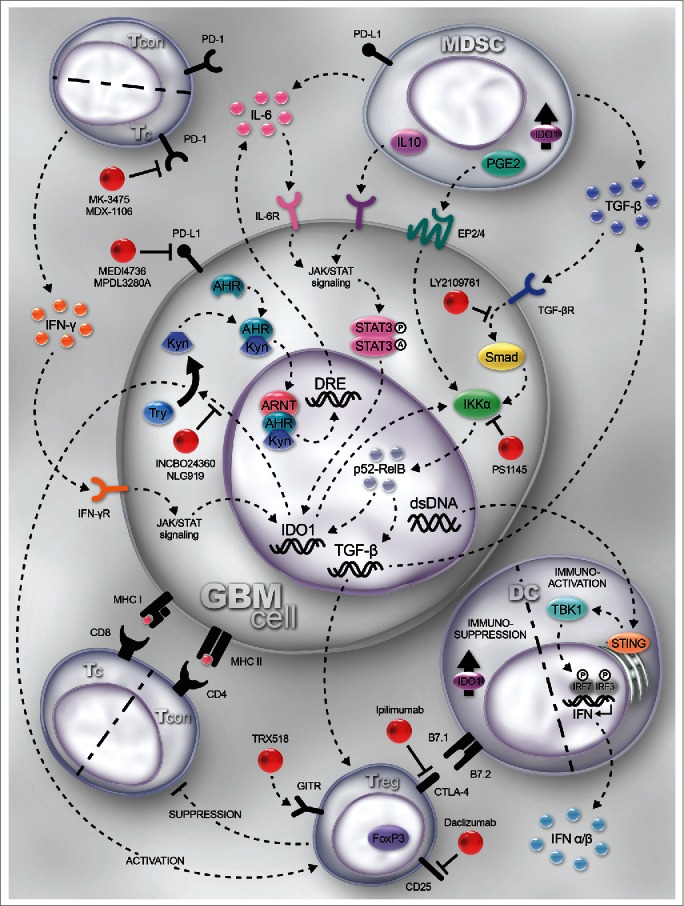
Mechanisms and immunotherapeutic targets for glioblastoma (GBM). GBM cells, tumor-resident dendritic cells (DC) and myeloid-derived suppressor cells (MDSC) express indoleamine 2,3 dioxygenase 1 (IDO1). IDO1 expression is regulated by the Jak/STAT and NF-κB pathways, which is induced by IFNγ- and TGF-β-receptor activation, respectively. IDO1 is a cytoplasmic enzyme that metabolizes tryptophan (Trp) to kynurenine (Kyn). Within the GBM cell, Kyn complexes with the aryl hydrocarbon receptor (Ahr), cytoplasmically, facilitating the nuclear translocation and further docking with aryl hydrocarbon receptor nuclear translocator (ARNT) to transcriptionally regulate IL-6, acting as an autocrine loop that amplifies and sustains IDO1 expression. Simultaneously, extracellular Kyn suppresses T effector responses while activating regulatory T cell (Treg; CD4^+^CD25^+^FoxP3^+^) function through a presumably overlapping mechanism. IDO1 directly activates NF-κB signaling which maintains and/or upregulates TGF-β expression. Increased TGF-β levels upregulate CTLA-4 and GITR expression by Treg. CTLA-4 interacts with B7.1 (CD80) and B7.2 (CD86) on DC, resulting in the induction of IDO1 (in DC) and commensurate downregulation of antigen presentation to T cells. Both GBM and MDSC express TGF-β, which synergizes with PD-L1 to suppress the T cell effector response via interaction with PD-1. Moreover, interleukin-10 (IL-10)- and prostaglandin E2 (PGE2)-expressing MDSC act on their cognate receptors expressed by GBM to ramify Jak/STAT and NK-κB-mediated signaling. DNA released by dead/dying GBM cells is phagocytized by resident DC to activate the STING pathway leading to Type 1 interferon (α/β) expression, supporting increased effectiveness of anti-GBM immunity. PD-1 is highly expressed by tumor-infiltrating cytotoxic T cells and PD-L1 is upregulated on cancer/stromal cells in response to T-cell-secreted IFNγ. Blocking the interaction of PD-1-expressing T cells with PD-L1 leads to increased effector function and enhanced GBM immunity. Targets for immunomodulation are shown in red. Note: Although IDO1 expression and signaling are shown in GBM cells, shared signaling patterns are presumed to be present in DC and MDSC as well. T_CON_: conventional CD4^+^FoxP3^−^ T cell; T_REG_: regulatory CD4^+^FoxP3^+^ T cell; T_C_: cytotoxic CD8^+^ T cell; INCBO24360/NLG919: inhibitors of IDO1; PS1145: inhibitor of the NF-κB pathway; TRX518: humanized monoclonal agonistic antibody for GITR; Ipilimumab: humanized monoclonal antibody for CTLA-4; LY2109761: TGF-β receptor kinase inhibitor; MK-3475/MDX-1106: humanized monoclonal antibodies to PD-1; MEDI4736/MPDL3280A: humanized monoclonal antibodies to PD-L1; Anti-Gr1: mSC-depleting antibody; Daclizumab: humanized anti-CD25 (IL-2Rα); STING: stimulator of interferon genes; TBK1: TANK-binding kinase 1; IRF3/7: interferon regulatory factor 3/7; STAT3: signal transducer and activator of transcription 3; A.^18,72,84-93^

The CNS was originally considered to be an immune-privileged site, in part, based on the superior growth of rat osteosarcoma cells that were intracranially injected into the brain compared to growth subcutaneously or intramuscularly.^4^ More recent observations indicate that the CNS is immunospecialized, based on the considerable interaction with the peripheral nervous system and the non-parenchymal ventricles, meninges and subarachnoid space.^5^ Inflammatory stimuli, including those induced by brain tumors, increase CNS immunogenicity due to microglial activation and blood–brain barrier (BBB) disruption.^6^ The latter occurs secondary to glioma cell invasion of the basement membrane.^7,8^ BBB disruption facilitates the drainage and presentation of CNS antigens to the cervical lymph nodes, which primes T cells for homing and infiltration to the tumor parenchyma. Interestingly, the pattern of leukocyte infiltration into GBM is not identical among tumors, with specific genetic subtypes including the mesenchymal profile, possessing higher levels of T cell infiltration.^9^ Coincidently, the mesenchymal subtype is almost universally observed in recurrent GBM after standard of care therapy.^10^ Commensurate to the inflammatory signals (i.e. cytokines, chemokines, growth factors) that brain tumors induce, are potently immunosuppressive mechanisms that include the tryptophan catabolic enzyme, indoleamine 2,3 dioxygensase 1 (IDO1). This rate-limiting enzyme is expressed in 96% of resected glioblastoma, with the upregulation correlating with a worse patient prognosis.^11,12^ IDO1 converts tryptophan into kynurenines, with the latter catabolite mediating inhibition/induction of apoptosis in effector T cells and/or amplification of immunosuppression by CD4^+^CD25^+^FoxP3^+^ regulatory T cells (Treg) (Fig. 1).^13^ Preclinically, tumor-derived IDO1 is essential for Treg accumulation and immunosuppression, since malignant brain tumors deficient for the enzyme result in spontaneous rejection mediated by a T-cell-dependent mechanism.^12^ Paradoxically, Treg incidence in newly diagnosed patient GBM is a neutral prognostic factor.^14^ Importantly, it has yet to be determined whether this finding holds true in recurrent GBM and this may be an important clinical consideration since our laboratory has experimental evidence from a model of spontaneously forming glioma suggesting that IDO1 functions differently in brain tumors depending on the newly diagnosed vs. recurrent context (unpublished observation). An alternative immunosuppressive pathway that contributes to T cell dysfunction is mediated by interactions between PD-1 and PD-L1, resulting in the loss of T cell effector function. Notably, both human GBM^15^ and tumor-infiltrating macrophages^16^ express high levels of PD-L1, suggesting the need for multi-cellular targeting for optimal immunotherapeutic benefit. Similar to other malignancies, cytotoxic T cells infiltrating GBM express high levels of PD-1.^17^ A third dominant and critical immunosuppressive pathway relevant to brain tumors is mediated by CTLA-4, which simultaneously inhibits effector T cell activation/proliferation and Treg activation/function in GBM.^18^ Interestingly, the interaction of CTLA-4 with dendritic cell (DC)-expressed B7, induces IDO1 expression.^19^ Thus, it will be interesting to determine whether co-inhibiting CTLA-4 and IDO1 lacks an additive/synergistic impact against brain tumors or if other undiscovered immunosuppressive mechanisms remain independent of the interaction.

### Therapeutic approaches

#### Vaccination

Therapeutic vaccination against cancer induces and/or rescues unproductive immune responses against tumor antigens intrinsically expressed or cross-presented by stromal cells.^20^ This immunity can be generated against mutated peptides,^21^ or post-translational modifications.^22^ To generate/rescue functional antitumor T cell responses, vaccines co-administer tumor peptide(s) and immuno-stimulatory adjuvant(s) to license DC for activating and expanding tumor-reactive T cells. Determining the optimal peptide(s) for targeting is a challenging task since many tumor-associated antigens are identified as “self” by the immune system.^23^ Given the shared neuroectodermal lineage of astrocytes and melanocytes, there is relatively significant overlap of shared tumor associated antigens between GBM and melanoma.^24^ This complicates targeting GBM with high specificity given the obvious potential for immunization against normal melanocytes.^24^ In practice, however, this phenomenon has not been observed in the majority of previously vaccinated GBM patients.^25^ Notably, *ex vivo* loading of a newly diagnosed GBM patient's DC with six GBM tumor-associated peptides can generate vaccine-specific immune responses that are not associated with an OS advantage.^26^ By vaccinating GBM patients with DC loaded with glioma-associated peptides combined with adjuvant poly-ICLC, approximately 60% of patients demonstrate glioma-associated immune responses, with <10 % of recurrent glioma patients demonstrating stable tumor regression.^27^ Overall, these studies highlight an important concept suggesting that, stimulating an immune response against exclusively tumor-associated peptides is not sufficient for controlling malignant progression in the majority of patients. 

Tumor neoantigens are considered to have higher potential for therapeutic vaccination. These neoantigens are generated during tumor evolution,^28^ often resulting in unique targets within individual patients.^23,28^ Some neoantigens, however, are present in a higher percentage of GBM, providing rational targets for focusing vaccination efforts against. One of the best characterized neoantigens is the epidermal growth factor receptor variant III (EGFRvIII), which is present in ∼20–30% of newly diagnosed GBM,^29^ carrying an independent negative prognosis for patients who survive >1  y after diagnosis.^30^ EGFRvIII is the result of an in-frame deletion leading to a new antigenic junction,^31^ capable of inducing both cellular and humoral immunity.^32^ Rindopepimut, a 13-amino acid EGFRvIII peptide vaccine conjugated to adjuvant, is currently utilized for targeting this neoantigen. Phase II EGFRvIII peptide vaccines have demonstrated vaccine immunogenicity and increased OS, with median at approximately 24 mo from diagnosis, compared to historical controls (Table 1).^32-34^ Survival advantage of treated patients correlate to the magnitude of induced tumor immunity, with tumor relapse occurring with loss of EGFRvIII expression based on immunohistochemical detection.^32-34^ While promising, these data could also indicate that, sensitivity to EGFRvIII detection by IHC is masked by patient-derived EGFRvIII antibodies or post-translational modification(s) as well as the independent loss due to radiation and/or chemotherapy.^35^ A two-arm randomized phase III trial (ACT IV) for recently diagnosed GBM is currently underway to better assess the efficacy of this approach (NCT01480479) (Table 2). With regard to targeting neoantigens in lower-grade glioma, mutant isocitrate dehydrogenase type 1 (IDH1) is carried by more than 70% of diffuse grade II and III gliomas,^36^ and targeting IDH1 by peptide vaccination has shown efficacy.^37^

**Table 1.** Completed clinical trials of immunotherapy for glioma.

Trial Name	Phase	Sample Size/Type of Glioma	New/Recurrent	TherapeuticModality	Primary and Secondary Endpoints	Result/Outcomes	Clinical Trial ID/Reference Number
**Dendritic cell (DC) vaccines**							
Immune response in patients with newly diagnosed glioblastoma multiforme treated with intranodal autologous tumor lysate-dendritic cell vaccination after radiation chemotherapy	Pilot	10	New	DC vaccine	PFS and OS	PFS: 9.5 mo OS: 28 mo	^94^
Integration of autologous dendritic cell-based immunotherapy in the primary treatment for patients with newly diagnosed glioblastoma multiforme: a pilot study	Pilot	8 (7 completed)	New	DC vaccine	PFS and OS	PFS at 6 mo: 75%, OS: 24 mo	^95^
Therapeutic vaccination against autologous cancer stem cells with mRNA-transfected dendritic cells in patients with glioblastoma	Pilot	11 (7 received DC vaccine)	New	DC vaccine against cancer stem cells	PFS and OS	PFS: 694 d, OS: 759 d	NCT00846456^96^
Dendritic cell vaccination in glioblastoma after fluoresence-guided resection	Pilot	5	New	DC vaccine	PFS and OS	PFS: 16.1 mo OS: 27.4 mo	^97^
α-type-1 polarized dendritic cell-based vaccination in recurrent high-grade glioma: a phase I clinical trial	I	9 (7 with GBM, 2 with WHO grade III) with HLA-A2 or A24 genotype	Recurrent	DC vaccine	SD and PD	1 SD (11%) 8 PD (89%)	^98^
Phase I trial of a multi-epitope-pulsed dendritic cell vaccine for patients with newly diagnosed glioblastoma	I	21 (17 new GBM, 3 recurrent GBM, 1 brainstem glioma)	New + Recurrent	multi-epitope-pulsed DC vaccine	PFS and OS	newly diagnosed: PFS: 16.9 mo OS: 38.4 mo	^26^
Dendritic cell vaccination combined with temozolomide retreatment: results of a phase I trial in patients with recurrent glioblastoma multiforme	I	14 (9 completed initial phase, 3 yield of DC vaccine was too low)	Recurrent	DC vaccine with pulsed autologous tumor cells previously exposed to TMZ *in vivo* + TMZ	OR and PFS	2 with OR 22% with 6-mo PFS	^99^
Gene expression profile correlates with T cell infiltration and relative survival in glioblastoma patients vaccinated with dendritic cell immunotherapy	I	23	New + Recurrent	DC vaccine + toll-like receptor agonists (imiquimod or poly-ICLC)	OS and survival rate	OS: 31.4 mo survival rates: 1 y (92%) 2 y (55%), 3 y (47%)	NCT00068510 ^9^
A phase I/II clinical trial investigating the adverse and therapeutic effects of a postoperative autologous dendritic cell tumor vaccine in patients with malignant glioma	I/II	17 (16 GBM, 1 WHO grade III)	New + Recurrent	DC vaccine	OS and survival rate	OS: 525 d, 5-y survival 18.8%	^100^
Induction of CD8^+^ T-cell responses against novel glioma-associated antigen peptides and clinical activity by vaccinations with α-type1 polarized dendritic cells and polyinosinic-polycytidylic acid stabilized by lysine and carboxymethylcellulose in patients with recurrent malignant glioma	I/II	22 (13 GBM, 5 anaplastic astrocytoma, 3 anaplastic oligodendroglioma, 1 anaplastic oligoastrocytoma). All with HLA-A2 genotype.	Recurrent	α-type 1 polarized DC with synthetic peptides for glioma-associated antigen epitopes + poly-ICLC	immune response and PFS	58% with positive immune response to at least one glioma-associated antigen, 9 (41%) with PFS at least 12 mo	^27^
Adjuvant immunotherapy with whole-cell lysate dendritic cells vaccine for glioblastoma multiforme: a phase II clinical trial	II	Randomized: 18 experimental vs. 16 control	New	DC vaccine + surgery + RT + chemo vs. surgery + RT + chemo	PFS, OS, and survival rates	PFS: 8.5 mo vaccine vs. 8.0 mo control (*p* = 0.075). OS: 31.9 mo vaccine vs. 15.0 mo control (*p* < 0.002). survival rates 1 y (88.9%) 2 y (44.4%), 3 y (16.7%) vaccine vs. 1 y (75.0%), 2 y (18.8%), and 3 y (0%) control	^101^
**EGFRvIII vaccines**							
A pilot study of IL-2Rα blockade during lymphopenia depletes regulatory T-cells and correlates with enhanced immunity in patients with glioblastoma	Pilot	Randomized: 3 experimental vs.3 control	New	EGFRvIII peptide vaccine +daclizumab (anti-IL-2Rα MAb)vs. vaccine + saline	safety and immune response	no autoimmune toxicity, decreased CD4+Foxp3+ Tregs with daclizumab	NCT00626015^102^
An epidermal growth factor receptor variant III-targeted vaccine is safe and immunogenic in patients with glioblastoma multiforme	I	12	New	*DC vaccine targeting EGFRvIII antigen	Time to progression (TTP) and OS	TTP from vaccination: 6.8 mo OS: 22.8 mo	^103^
Immunologic escape after prolonged progression-free survival with epidermal growth factor receptor variant III peptide vaccination in patients with newly diagnosed glioblastoma	II	18	New	EGFRvIII peptide vaccine	PFS, OS, and immune response	6-mo PFS was 67% after vaccinationand 94% after diagnosis.OS: 26.0 mo,significantlylonger than matched cohort (*p* = 0.0013).Development of specific antibody (*p* = 0.025) or delayed-type hypersensitivity (*p* = 0.03) had significant effect on OS	^32^
Greater chemotherapy-induced lymphopenia enhances tumor-specific immune responses that eliminate EGFRvIII-expressing tumor cells in patients with glioblastoma	II	22	New	EGFRvIII peptide vaccine with either standard-dose or dose-intensified (DI) TMZ	PFS, OS, and immune response	PFS 15.2 mo OS: 23.6 mo Both humoral and cellular vaccine-induced immune responses are enhanced by DI TMZ	^34^
A phase II, multi-center trial of rindopepimut (CDX-110) in newly diagnosed glioblastoma: the ACT III study	II	65	New	Rindopepimut (CDX-110)	PFS and OS	PFS at 5.5 mo was 66% (approximately 8.5 mo from diagnosis). OS: 21.8 mo. 36-mo OS was 26%	^33^
**Heat-shock protein (HSP) vaccines**							
Pilot study of intratumoral injection of recombinant heat shock protein 70 in the treatment of malignant brain tumors in children	Pilot	12 (2 GBM, 2 astrocytoma, 3 anaplastic astrocytoma, 2 anaplastic ependymoma, 1 choroid plexus carcinoma, 1 primitive neuroectodermal tumor, 1 B-cell non-Hodgkin's lymphoma).	New	HSP 70 vaccine	CR and PR	1 CR (8%) 1 PR (8%)	^104^
Heat-shock protein peptide complex-96 vaccination for recurrent glioblastoma: a phase II, single-arm trial	II	41	Recurrent	HSPPC-96 vaccine	OS and survival rate	OS: 42.6 weeks.90.2% alive at 6 mo29.3% alive at 12 mo 27 (66%) lymphopenic prior to therapy leading to decrease OS	^25^
**Other peptide vaccines**							
Wilms tumor 1 peptide vaccination combined with temozolomide against newly diagnosed glioblastoma: safety and impact on immunological response	I	7	New	Wilms tumor 1 peptide vaccination	PFS	All patients still alive at time of study publication. PFS: 5.2–49.1 mo	^105^
**Viral vaccines**							
Phase IB study of gene-mediated cytotoxic immunotherapy adjuvant to up-front surgery and intensive timing radiation for malignant glioma	IB	13 (12 completed therapy)	New	Adenoviral vector with herpes simplex virus thymidine kinase gene + valacyclovir	Survival rate	33% alive at 2 y and 25% alive at 3 y	^106^
**Autologous vaccines**							
First clinical results of a personalized immunotherapeutic vaccine against recurrent, incompletely resected, treatment-resistant glioblastoma multiforme (GBM) tumors, based on combined allo- and auto-immune tumor reactivity	Pilot	9	Recurrent	Gliocav (ERC 1671) composed of autologous + allogeneic antigens + GM-CSF + low-dose cyclophosphamide	OS	OS: 100% alive at 26 weeks, 77% alive at 40 weeks	^107^
Phase I trial of a personalized peptide vaccine for patients positive for human leukocyte antigen–A24 with recurrent or progressive glioblastoma multiforme	I	12 (all positive for HLA-A24)	Recurrent	ITK-1 peptide vaccine	safety and immune response	No serious adverse drug reactions. Dose-dependent immune boosting	^108^
Phase I/IIa trial of autologous formalin-fixed tumor vaccine concomitant with fractionated radiotherapy for newly diagnosed glioblastoma. Clinical article	I/IIa	24 (2 excluded from final analysis)	New	Autologous formalin-fixed vaccine	PFS, OS, and survival rate	PFS: 7.6 mo OS: 19.8 mo 40% alive at 2 y	^109^
Phase I/IIa trial of fractionated radiotherapy, temozolomide, and autologous formalin-fixed tumor vaccine for newly diagnosed glioblastoma	I/IIa	24	New	Autologous formalin-fixed tumor vaccine	PFS, OS, and survival rates	33% with PFS ≥ 24 mo. PFS: 8.2 mo OS: 22.2 mo. 47% alive at 2 y, 38% alive at 3 y	^110^
**Adoptive T cell therapy**							
Autologous T cell therapy for cytomegalovirus as a consolidative treatment for recurrent glioblastoma	I	19 (13 with successfully expanded CMV-specific T cells)	Recurrent	CMV-specific T cells	OS, PFS, and molecular profiling	OS: 403 d [range 133–2,428 d]. PFS: >35 weeks [range 15.4–254 weeks]. 4 of 10 who completed T cell therapy remained progression free during study period. Distinct gene expression signatures to CMV-specific T cell therapy correlated with clinical response.	^67^

Sample size/type of glioma indicate GBM unless otherwise noted.

Results/outcomes indicate median unless otherwise noted.

Trials were identified on pubmed with the search terms: “glioblastoma” AND “patients” AND “trial,” between the years, 2010 and 2015.

**Table 2.** Ongoing trials of immunotherapy for glioma and brain metastases.

Trial Name	Phase	Target accrual	Location	New/ Recurrent/ Metastatic	TherapeuticModality	Primary and Secondary Endpoints	Clinical Trial Identifier
**DC vaccine**							
Study of a drug [DCVax®-L] to treat newly diagnosed GBM brain cancer	III	300	Multi-center	New	DCVax®-L (DC vaccine)	OS, PFS	NCT00045968
**EGFRvIII vaccine**							
An International, Randomized, Double-Blind, Controlled Study of Rindopepimut/GM-CSF With Adjuvant Temozolomide in Patients With Newly Diagnosed, Surgically Resected, EGFRvIII-positive Glioblastoma	III	700	Multi-center	New	Rindopepimut/GM-CSF	OS, PFS, safety and tolerability	NCT01480479
**Heat-shock protein (HSP) vaccine**							
A Phase II Randomized Trial Comparing the Efficacy of Heat Shock Protein-Peptide Complex-96 (HSPPC-96) (NSC #725085, ALLIANCE IND # 15380) Vaccine Given With Bevacizumab vs. Bevacizumab Alone in the Treatment of Surgically Resectable Recurrent Glioblastoma Multiforme (GBM)	II	222	Northwestern University	Recurrent	HSPPC-96 + Bevacizumab vs. Bevacizumab	OS, PFS, adverse events	NCT01814813
**STAT3 inhibitor**							
A Phase I Trial of WP1066 in Patients With Central Nervous System (CNS) Melanoma and Recurrent Glioblastoma Multiforme (GBM)	I	21	M.D. Anderson	Recurrent	WP1066	maximum tolerated dose (MTD), dose-limiting toxicity (DLT)	NCT01904123
**Immune checkpoint blockade**							
Phase I Study of Ipilimumab, Nivolumab, and the Combination in Patients With Newly Diagnosed Glioblastoma	I	42	NRG Oncology (PA)	New	Ipilimumab and/or Nivolumab + TMZ	immune-related DLTs, adverse events, biomarker analysis of immune cells, survival rate	NCT02311920
Phase II Study of Pembrolizumab (MK-3475) With and Without Bevacizumab for Recurrent Glioblastoma	II	79	Dana-Farber Cancer Institute, Massachusetts General Hospital	Recurrent	Pembrolizumab +/− Bevacizumab	PFS, MTD, safety, tolerability, OS, overall radiographic response	NCT02337491
Phase 2 Study to Evaluate the Clinical Efficacy and Safety of MEDI4736 in Patients With Glioblastoma (GBM)	II	84	Multi-center	New + Recurrent	MEDI4736 +/− Bevacizumab	OS, PFS, adverse events, ORR, pharmokinetic profile, quality of life	NCT02336165
A Randomized Phase 3 Open Label Study of Nivolumab vs. Bevacizumab and Multiple Phase 1 Safety Cohorts of Nivolumab or Nivolumab in Combination With Ipilimumab Across Different Lines of Glioblastoma	III	440	Multi-center	Recurrent	Nivolumab, Nivolumab + Ipilimumab, Bevacizumab	safety, tolerability, OS, PFS, ORR	NCT02017717
**Adoptive T cells**							
Pilot Study of Autologous T Cells Redirected to EGFRVIII-With a Chimeric Antigen Receptor in Patients With EGFRVIII+ Glioblastoma	I	12	University of Pennsylvania, UCSF	New + Recurrent	CAR T cells to EGFRvIII	number of adverse events	NCT02209376
Evaluation of Recovery From Drug-Induced Lymphopenia Using Cytomegalovirus-specific T cell Adoptive Transfer	I	12	Duke University	New	CMV-autologous lymphocyte transfer	T cell response, safety	NCT00693095
Administration of HER2 Chimeric Antigen Receptor Expressing CMV-Specific Cytotoxic T Cells In Patients With Glioblastoma Multiforme (HERT-GBM)	I	16	Baylor College of Medicine	Recurrent	CMV-specific Cytotoxic T Lymphocytes	DLT, safety with increasing doses, tumor size	NCT01109095
Phase I Study of Cellular Immunotherapy Using Central Memory Enriched T Cells Lentivirally Transduced to Express an IL13Rα2-Specific, Hinge-Optimized, 41BB-Costimulatory Chimeric Receptor and a Truncated CD19 for Patients With Recurrent/Refractory Malignant Glioma	I	44	City of Hope Medical Center	Recurrent + Refractory	Enriched T cells expressing IL13Rα2	toxicity, DLT, change in tumor length, cytokine levels, PFS, OS, quality of life, T cell detection in tumor, IL13Ra2 antigen expression level	NCT02208362
A Phase I/II Study of the Safety and Feasibility of Administering T Cells Expressing Anti-EGFRvIII Chimeric Antigen Receptor to Patients With Malignant Gliomas Expressing EGFRvIII	I/II	160	National Institutes of Health	Recurrent	CAR T cells to EGFRvIII	safety, PFS, *in vivo* survival of CAR cells, radiographic changes after treatment	NCT01454596
**Brain metastasis**							
Ipilimumab Induction in Patients With Melanoma Brain Metastases Receiving Stereotactic Radiosurgery	II	40	University of Michigan	Metastatic	Ipilimumab	local control rate, toxicity rate, overall survival rate, intracranial response rate, time to event	NCT02097732
A Multi-center, Single Arm, Phase 2 Clinical Study on the Combination of Radiation Therapy and Ipilimumab, for the Treatment of Patients With Melanoma and Brain Metastases	II	66.	Multi-center	Metastatic	WBRT 30 Gy in 10 fractions + Ipilimumab	1-y survival rate, PFS (intracranial and extracranial), OS, response rate, adverse event rate	NCT02115139
A Phase II Study of Nivolumab and Nivolumab Combined With Ipilimumab in Patients With Melanoma Brain Metastases	II	75	Melanoma Institute Australia	Metastatic	Nivolumab vs. Nivolumab + Ipilimumab	CR, PR, PFS(intracranial and extracranial), overall response rate, OS, safety and tolerability, quality of life, immune response, tissue and blood biomarkers, FET-PET response	NCT02374242
A Multi-Center Phase 2 Open-Label Study to Evaluate Safety and Efficacy in Subjects With Melanoma Metastatic to the Brain Treated With Nivolumab in Combination With Ipilimumab Followed by Nivolumab Monotherapy	II	148	The Angeles & Clinic Research Institute,St. Luke's Hospital & Health Network (PA)	Metastatic	Nivolumab + Ipilimumab followed by Nivolumab	CR and PR (intracranial and extracranial), OS, safety, tolerability	NCT02320058

Clinical trials were identified on the website clinicaltrials.gov as of 05/2015.

To address tumor relapse from generation of antigenic variants in the process of targeting a single peptide, alternative vaccine approaches have been created to target a broad range of antigens, simultaneously. One exciting approach utilizes heat shock protein (HSP) peptide complexes (HSPPC-96) derived from a GBM patient's resected tumor. Intracellular HSP physiologically binds peptides with extracellular HSP capable of mediating the internalization of HSPPC-96 into APCs for efficient MHC-I and MHC-II presentation of tumor peptides.^38,39^ Clinically, HSPPC-96 vaccine generates a tumor-reactive T cell response.^39^ In a phase II, single arm trial for surgically resectable recurrent GBM, HSPPC-96 vaccine increased the median OS to an impressive 42.6 weeks, which provides a substantial survival benefit when compared to historical controls.^25^ Interestingly, a predictor of poor response to vaccination was lymphopenia at the time of vaccination, a side effect likely attributable to previous chemotherapy, radiation and/or decadron administration.^25^ An alternative approach to targeting multiple epitopes, simultaneously, is utilizing pulsed autologous DC with tumor lysate. This approach, identified as DCVax®-L, is currently in a Phase III trial for patients with newly-diagnosed GBM (NCT00045968).

 Over the past 3 y, technological advances and clinical discoveries have sparked the development of next-generation vaccines. The first observation from both preclinical subcutaneous fibrosarcoma and clinical melanoma studies demonstrated that CD8^+^ T cells responsible for eradicating tumors must recognize tumor-specific peptides that have high affinity for MHC-I.^40-42^ In preclinical subcutaneous fibrosarcomas, peptide affinity determines whether a peptide can be cross-presented by tumor-associated macrophages and thereby serve to optimally stimulate T cells to produce high levels of cytokine in the tumor microenvironment.^40^ Recent technological advances now facilitate these “rejection” antigens to be reliably identified using (i) genome-wide exomic sequencing to find mutations and (ii) peptide affinity algorithms to identify peptides with high peptide–MHC affinity.^41,43^ This approach has been validated preclinically demonstrating that, vaccinating against a model “rejection peptide” achieves tumor eradication of aggressive melanoma.^21,44^ Creating personalized vaccines to target these predicted rejection antigens is now recognized as a promising approach against non-CNS tumors and should be studied with regard to whether similar efficacy is achievable against aggressive tumors in the CNS. 

#### Checkpoint blockade

 Over the past 15–20 y, it has become recognized that inhibitory receptors on T cells play an important role in suppressing T-cell-mediated antitumor responses.^45^ These inhibitory receptors are referred to as immune checkpoints due to their role in preventing inappropriate/prolonged activation. To date, the checkpoints that have been targeted with the most impressive clinical antitumor responses are CTLA-4 and PD-1. During CD8^+^ T cell activation, CTLA-4 is upregulated and inhibits further T cell activation and proliferation.^46^ CTLA-4 is also expressed on CD4^+^ T cells where it functions to enhance Treg-mediated immunosuppression.^47^ Ipilimumab, a humanized CTLA-4 antibody, was the first FDA-approved immune checkpoint inhibitor. Much clinical experience with ipilimumab has been in treating metastatic melanoma, in which there is an approximately 2% complete response rate that remains durable.^48^ Responses have been observed against both non-CNS and CNS-infiltrated melanoma metastases.^49^ Preclinically, mice bearing intracranial glioma and treated with CTLA-4 mAb (clone 9H10) develop robust antitumor immunity without affecting Treg function.^18^ Clinically, the administration of ipilimumab for GBM has been limited to a small number of GBM patients in the recurrent setting.

More recently, efforts aimed at inhibiting the PD-1/PD-L1 pathway have generated significant interest. Tumor-infiltrating lymphocytes express high levels of PD-1 in most cancers, including GBM,^17^ as a result of chronic antigen stimulation by the tumor.^50^ When PD-1-expressing T cells interact with PD-L1, T cell effector function is inhibited.^50^ PD-L1 is upregulated in GBM through the following mechanisms: (i) oncogenic signaling as a result of PTEN loss,^15^ (ii) paracrine signaling,^16^ and/or (iii) “adaptive immune resistance” whereby T-cell-secreted IFNγ induces PD-L1 expression on neighboring cells.^51^ While clinical trials studying PD-1 and PD-L1 blockade are currently recruiting patients for GBM (NCT02337491, NCT02336165), the effectiveness of this approach has been characterized in treating refractory melanoma, providing an objective response rate (ORR) of approximately 15–30% as monotherapy with complete responses restricted to <6 % of patients.^52,53^ Since PD-1/PD-L1 does not induce T cell infiltration into tumors, but rather rescues/prevents T cell anergy, it is not surprising that patients associated with the best responses possess higher tumor-infiltrating T cell levels prior to treatment that is co-localized with PD-L1 expression.^54^

The most promising outcomes related to immune checkpoint inhibition have been achieved through combinatorial CTLA-4/PD-(L)1 blockade,^55-57^ which is consistent with these pathways providing non-redundant T cell inhibition. In a recent randomized control trial for untreated advanced melanoma, dual CTLA-4 and PD-1 blockade provided an improved ORR (58%) compared to monotherapy CTLA-4 (19%) and monotherapy PD-1 (44%).^57^ Interestingly, dual CTLA-4 and PD-1 blockade was found to be superior compared to PD-1 monotherapy in treating PD-L1-negative tumors, but not PD-L1-positive tumors, suggesting that CTLA-4 blockade induces T cell infiltration into tumors.^57^ Consistent with these findings in melanoma, preclinical models of GBM demonstrate high rates of survival when treated with simultaneous PD-L1 and CTLA-4 blockade, as compared to the respective monotherapies.^58^ Clinically, trials aimed at GBM patient treatment with ipilimumab and nivolumab (humanized PD-1 mAb) are already underway (NCT02311920, NCT02017717). In addition, several clinical trials enrolling patients with brain metastases are also in progress, including studies using PD-1 mAb alone and CTLA-4 combined with PD-1 mAb (NCT02374242, NCT02320058). 

In addition to PD-1 and CTLA-4, therapeutic modulation of other immune inhibitory and stimulatory pathways is currently being evaluated preclinically and in early-phase trials (Table S1). Blocking inhibitory receptors LAG-3 or TIM-3 in combination with PD-1 blockade provides impressive preclinical tumor control in non-CNS tumor models ^59,60^. Dual LAG-3 and PD-1 blockade is currently being tested against multiple non-CNS solid tumors in a Phase I trial (NCT01968109). Modulating both inhibitory and stimulatory immune pathways may also be a promising approach as dual CTLA-4 blockade and ICOS stimulation provides improved antitumor control against preclinical murine melanoma and prostate cancer.^61^ This strategy may also be effective in GBM, as triple therapy with RT combined with CTLA-4 inhibition and 41BB stimulation provides improved tumor control compared to each dual therapy.^62^

#### Adoptive T cell therapy

Previously described therapeutic approaches endeavor to rescue or induce endogenous T cell responses, while adoptive T cell therapy provides an alternative strategy that involves expanding tumor-specific autologous T cells, *ex vivo*, followed by venous infusion into the same individual. Tumor-reactive T cells are isolated from (i) peripheral blood, (ii) surgically resected tissue or (iii) generated by transduction of the patient's autologous T cells with vectors encoding T cell receptors (TCR) or chimeric antibody receptors (CAR).^63^ The capacity of adoptive T cell therapy to eradicate a large established tumor burden has been demonstrated with the re-infusion of tumor-infiltrating lymphocytes specific to melanoma,^64^ as well as CAR-based treatment for CD19^+^ B-cell malignancies.^65^

In GBM patients, adoptive T cell therapy has been used to target human cytomegalovirus (CMV) antigens expressed by tumor cells.^66-68^ A recent study treating 11 recurrent GBM patients with infusions of autologous adoptively transferred CMV-specific T cells led to a median OS of >57  weeks, with four patients remaining progression-free throughout the study period.^67^ Longer progression-free survival (PFS) was associated with decreased expression of checkpoint receptors on T cells suggesting that, maintaining effector function of adoptively transferred T cells is required for a durable clinical response.^67^ A clinical trial investigating CMV adoptive T cell therapy is ongoing (NCT00693095). 

Utilizing CAR T cell adoptive therapy for GBM patients is a logical ‘next step’ for autologous therapy. CAR consist of an extracellular antibody domain fused to a T cell cytoplasmic signaling domain. Preclinical glioma CAR studies targeting HER2 and the previously described EGFRvIII reported impressive results.^69,70^ Clinical trials targeting both antigens are ongoing (NCT02209376, NCT01109095, NCT01454596), as well as a CAR trial targeting IL13Rα2 (NCT02208362). Future studies should focus on identifying additional tumor-specific antigenic targets shared among patients and/or developing an approach to personalize CAR technology to each patient's tumor antigen profile.

#### Combination approaches

 Optimal immunotherapy approaches must provide immune activation while, simultaneously, countering inhibitory checkpoint blockade signals. Moreover, it is now recognized that single modality immunotherapy has limitations that can be overcome by multi-targeted strategies. Some of the promising immunotherapeutic combinations will be further discussed.

### Radiation, DNA sensors and immune checkpoint blockade

 Combining ablative radiation with immune checkpoint blockade is a promising immunotherapeutic combination. While radiation was previously viewed as immunosuppressive, preclinical tumor models have demonstrated that hypofractionated ablative radiation can generate tumor regression that is T cell dependent.^71^ The mechanism accounting for this effect likely relies on: (i) radiation-induced tumor inflammation and cell death, (ii) DC that phagocytize “released” cancer cell DNA capable of activating the Stimulator of IFN genes (STING) pathway, (iii) increased type 1 IFN-licensed DC that prime tumor-specific T cells and (iv) reactive T cells that home to and engage the tumor with strong effector function.^72^ Type I IFN appears to be essential for antitumor immunity, with intratumoral injection of a STING agonist significantly improving tumor control following radiation in experimental models.^72^ While the impact of combined radiation and STING activation has yet to be confirmed in CNS tumor models, it is notable that immune-mediated control of glioma outgrowth is dependent on STING-mediated induction of type 1 IFN.^73,74^ Accordingly, glioma patient prognosis is dictated, in part, by type 1 IFN single nucleotide polymorphisms (SNPs).^75^ Collectively, these findings suggest that immune-modulating approaches utilizing a combination of RT and STING agonists may be promising to combat tumors in the CNS.

 For both CNS- and non-CNS-resident tumors, combined RT and immune checkpoint blockade has demonstrated increased effectiveness when compared to RT alone. In a mouse orthotopic glioma model, combining radiation with anti-PD-1 provides an additive effect that improves OS, when compared to either therapy administered individually.^76^ As a mechanism accounting for the enhanced effectiveness of combinatorial treatment, radiation-induced inflammation results in PD-L1 upregulation on cancer cells, macrophages and DC.^77^ Similarly, combinatorial anti-CTLA-4 and RT leads to tumor control in a preclinical model of breast cancer.^78^ Notably, the latter combination has thus far yielded a less impressive impact on OS when compared to combinatorial RT and PD-(L)1 blockade.^77^ More recently, it was reported that control of preclinical melanoma is optimal when simultaneously treating with RT, anti-PD-(L)1 and anti-CTLA-4, when compared to dual therapy.^79^ Each modality induced a unique immune activating profile with RT expanding the TCR repertoire, anti-CTLA-4 inhibiting Treg function and increasing the Tc/Treg ratio and anti-PD-(L)1 preventing T cell exhaustion/dysfunction in tumors.^79^ Interestingly, RT combined with anti-CTLA-4 and anti-4-1BB induces similar antitumor activity, with the latter agonist causing direct stimulation to cytolytic T cells, resulting in an increased level of survival and T cell infiltration when compared to dual therapy.^62^

Clinically, combining RT and checkpoint blockade was recently tested for the first time in a phase I trial. Patients received three doses of hypofractionated radiation to a single metastatic melanoma lesion followed by anti-CTLA-4 treatment. While median OS was <11  mo, local tumor control was achieved in the irradiated lesions for all 12 patients analyzed.^79^ Although CNS metastases were not targeted in this trial, local tumor control of melanoma brain metastases has been reported in a case series using both whole-brain RT (30 Gy/10 fractions) and stereotactic RT (20–24 Gy/1 fraction) for patients who received RT following a course of ipilimumab.^80^ Based on the strong promise of radiation combined with checkpoint blockade to achieve local tumor control in CNS and non-CNS tumors, future preclinical and clinical GBM studies should investigate how to optimize this approach. For melanoma brain metastases, two phase II trials combining RT approaches with ipilimumab for brain metastases are currently underway (NCT02115139, NCT02097732).

### Vaccination and immune checkpoint blockade

Therapeutic vaccination may fail if the strategy does not optimally expand tumor-reactive T cells and/or vaccine-generated T cells lose effector function in the immunosuppressive tumor microenvironment.^81^ PD-1/PD-L1 interactions likely dampen vaccine responses by two mechanisms: (i) in the draining lymph node where vaccine adjuvant-induced inflammation results in PD-L1 expression on antigen-presenting cells that inhibits maximal expansion of vaccine-generated T cells,^82^ and (ii) in the tumor itself whereby “adaptive immune resistance” ^51^ is generated by T cells secreting IFNγ that induces PD-L1 upregulation on neighboring cells leading to T cell anergy. Thus, combining vaccination with PD-1/PD-L1 antibody blockade is likely to provide a synergistic effect. In support of this, long-established preclinical melanomas resistant to dual PD-L1 and CTLA-4 blockade are eradicated by vaccination in approximately 33% of mice, but eradicated by vaccination combined with anti-PD-L1 in 80% of mice.^21^ In an independent preclinical study of subcutaneous tumors, vaccination combined with PD-(L)1 and CTLA-4 inhibition led to improved tumor rejection and mouse survival, when compared to dual- and mono-therapeutic treatment.^83^ Clinically, the combination of peptide vaccination and PD-1 blockade is currently being evaluated in patients diagnosed with melanoma (NCT01176474). Since the majority of prior studies have been performed in non-CNS tumor models, future preclinical and clinical studies should evaluate these treatment approaches in patients with GBM.

## Conclusions

GBM is a highly immunosuppressive tumor that is refractory to traditional therapies and difficult to treat based on its anatomical location. Metastatic tumors in the brain, with a prevalence of >20 :1 compared to GBM, also present much treatment challenge. Past immunotherapeutic efforts for brain tumors have predominantly focused on therapeutic vaccination that has achieved promising immune activity and clinical responses. However, durable responses remain rare highlighting the need to further test existing promising approaches including gene therapy (supplemental text, Table S2), develop next-generation therapeutics (i.e. IDO inhibitors/STING agonists,) and test novel immunotherapeutic combinations (Table 3). Because antitumor immune responses occur in the context of inflammation, the possibility for tumor- and therapy-induced inflammation to cause additive/synergistic brain swelling and neurologic compromise must be recognized. While Decadron is routinely used to counter brain swelling, its use is restricted to low doses in immunotherapeutic trials as it is also extremely immunosuppressive. Next-generation CNS immunotherapies, if more efficacious, may carry an even higher risk for brain swelling and neurological compromise, thus identifying non-immunosuppressive anti-inflammatory approaches is important. Utilizing bevacizumab, a VEGF neutralizing antibody that secondarily decreases inflammation, is one such approach currently being explored in combination with GBM immunotherapy (NCT02336165, NCT01814813). CNS immunotherapy has a bright future in this current “golden age” of immunotherapy . Future studies should focus on providing patients with this battery of ever-evolving options, while also recognizing that CNS malignancies have unique immunosuppressive phenotypes that need to be specifically targeted.

**Table 3.** High priority questions for increasing immunotherapeutic efficacy against tumors in the CNS.

Preclinical	
• Do inhibitors that co-target IDO1 and IDO2 provide superior efficacy when compared to monotherapy? • Will inhibitors of tryptophan catabolism complement other immunotherapies? • Which capacity of IDO1 is more important for immunotherapeutic efficacy: signal transduction modifier vs. tryptophan catabolism? • What is the best approach for further identification of ubiquitous GBM-specific neoantigens for translation into vaccine and/or adoptive T cell therapeutic approaches? • Is there an optimal vaccination approach to generate functional T cell responses and is this GBM subtype-specific (i.e. responsiveness in classical vs. mesenchymal, newly diagnosed vs. recurrent)? • Do different GBM subtypes possess correlative mutational frequencies that associate with responsiveness to immunotherapy? • Will survival outcomes be enhanced with combinatorial approaches (vaccine ± RT ± checkpoint blockade ± STING activation)?	
Clinical	
• Will GBM respond to immune checkpoint blockade? • What is the best approach for identifying patient cohorts that will benefit from immunotherapy? • What is the best approach for monitoring treatment effectiveness in GBM patients to immunotherapy (i.e. peripheral blood markers, tryptophan metabolic profiling or IHC markers in the tumor)? • What is the best approach to limit brain swelling following immunotherapy? Is bevacizumab an alternative to decadron that can be easily added without defusing effectiveness?	

## Supplementary Material

1082027_supplemental_files.zip
