# Supplementary material for: Immunotherapy for cancer in the central nervous system: Current and future directions
Source: Oncoimmunology. 2015 Sep 11;5(2):e1082027. doi: 10.1080/2162402X.2015.1082027 (PMC4801467; doi:10.1080/2162402X.2015.1082027)
Supplement: 1082027_supplemental_files.zip [file koni-05-02-1082027-s001.zip › 1082027 supplemental files/Supplemental #1.pptx]

## Slide 1
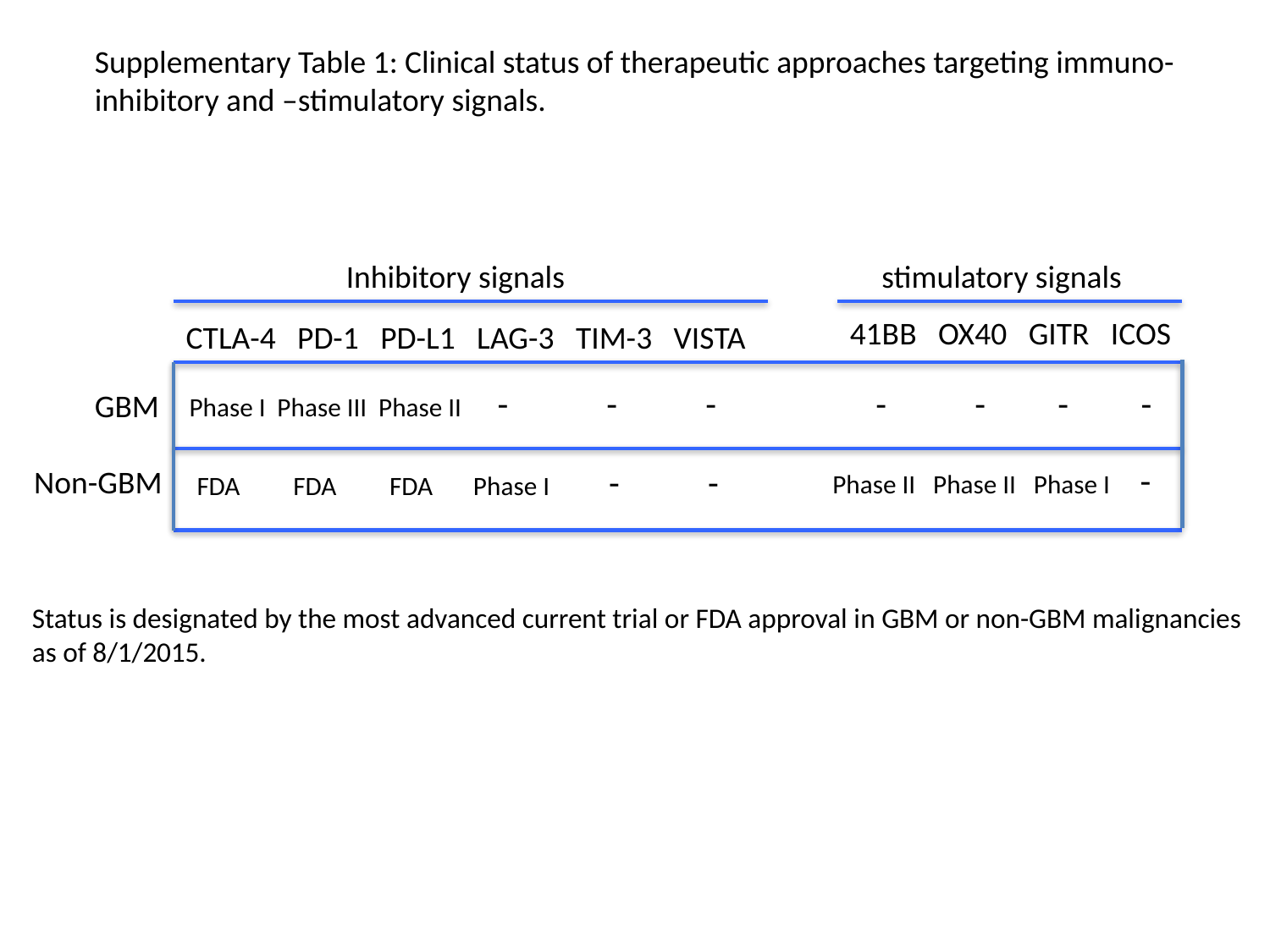

Supplementary Table 1: Clinical status of therapeutic approaches targeting immuno-inhibitory and –stimulatory signals.
Inhibitory signals
stimulatory signals
41BB OX40 GITR ICOS
CTLA-4 PD-1 PD-L1 LAG-3 TIM-3 VISTA
Phase I Phase III Phase II - - -
- - - -
GBM
Phase II Phase II Phase I -
FDA FDA FDA Phase I - -
Non-GBM
Status is designated by the most advanced current trial or FDA approval in GBM or non-GBM malignancies
as of 8/1/2015.
