# Supplementary material for: Immunotherapy for cancer in the central nervous system: Current and future directions
Source: Oncoimmunology. 2015 Sep 11;5(2):e1082027. doi: 10.1080/2162402X.2015.1082027 (PMC4801467; doi:10.1080/2162402X.2015.1082027)
Supplement: 1082027_supplemental_files.zip [file koni-05-02-1082027-s001.zip › 1082027 supplemental files/Supplemental #2.docx]

| **Trial Name** | **Phase** | **Therapy** | **Identifier** |
| --- | --- | --- | --- |
| A phase I study of Ad-RTS-hIL-12, an inducible adenoviral vector engineered to express hIL-12 in the presence of the activator ligand veledimex in subjects with recurrent or progressive glioblastoma or grade III malignant glioma | I | AdV engineered to express hIL-12 (INXN-2001) | NCT02026271 |
| A phase I study of M032 (NSC 733972), a genetically engineered HSV-1 expressing IL-12, in patients with recurrent/progressive glioblastoma multiforme, anaplastic astrocytoma, or gliosarcoma | I | Oncolytic HSV-1 expressing IL-12 (M032) | NCT02062827 |
| A non-randomized, open-label dose-finding trial of combined cytotoxic and immune-stimulatory strategy for the treatment of resectable primary malignant glioma | I | Ad-hCMV-TK and Ad-hCMV-Flt3L | NCT01811992 |
| Phase I trial of a measles virus derivative producing CEA (MV-CEA) in patients with recurrent glioblastoma multiforme (GBM) | I | MV-CEA | NCT00390299 |

**Supplementary Table 2: Ongoing immunotherapy trials using gene therapy**
