# Supplementary material for: Immunotherapy for cancer in the central nervous system: Current and future directions
Source: Oncoimmunology. 2015 Sep 11;5(2):e1082027. doi: 10.1080/2162402X.2015.1082027 (PMC4801467; doi:10.1080/2162402X.2015.1082027)
Supplement: 1082027_supplemental_files.zip [file koni-05-02-1082027-s001.zip › 1082027 supplemental files/Supplemental Discussion.docx]

**Supplemental Discussion**

Gene therapy-based approaches utilizing viral-mediated transduction of -cancer, -stromal and/or -stem cells for the delivery of cytotoxic factors to glioma has been in-progress for greater than 20 years (1). Moreover, given the early *in vitro* studies demonstrating that exogenous administration of interleukin-12 (IL-12) stimulates antitumor effects against malignant glioma cells (2), subsequent work targeting intracranial glial tumors provided additional evidence that glioma cells could be transduced to express the Th1-associated cytokine commensurate with immune-mediated tumor rejection (3). Similarly, injection of a glioma cell line expressing the T cell trophic cytokine, IL-2, combined with exogenous recombinant IL-12, led to further effectiveness at inducing the regression of established intracranial brain tumors in rodent models. The immunotherapeutic effect conveyed by adenoviral transduction of glioma cells was not exclusive to one type of viral delivery vehicle, since herpes simplex- (4), vaccinia- and semliki forest (5)-virus also resulted in the induction of immune-mediated antitumor effects (6). Combined with the innate immune-activating features of viral delivery (7), these early results suggested that gene therapy was a promising new approach for inducing a potent immune response to established intracranial tumors.

Aside from the focus on IL-2/-12, several additional targets have been shown to regulate the immune response against brain tumors after viral delivery of IL-4 (8), the NKG2D ligand, MICA (9), B7.1 (10), as well as mesenchymal stem cells transduced to express IL-18 (11). More recent studies have examined the mechanism of immunosuppression that otherwise disables gene therapeutic approaches. Curtin *et al*. demonstrated that, while the intratumoral delivery of adenoviral vectors (Ad) expressing Fms-like Tyrosine Kinase 3 ligand (Flt3L) and herpes simplex type 1-thymidine kinase (TK) with ganciclovir (+GCV) elicits T cell-dependent tumor regression, the addition of a Treg-neutralizing/depleting antibody disables the antitumor immune response by simultaneously inhibiting tumor-specific effector T cells in a mouse glioblastoma multiforme (GBM) model (12). Interestingly, further study indicated that this effect was mediated, in-part, through a TLR2-dependent release of HMGB1 (13). Notably, the preclinical application of gene therapy-mediated immunomodulation against brain tumors is not restricted to rodent models, as this approach was recently shown to be effective in a canine case study (14).

Currently, several clinical trials utilizing gene therapeutic approaches that elicit and/or modulate tumor immunity are in-progress (Supplementary Table 1). This includes NCT02062827, which utilizes a single dose of HSV-1 (M032) infused through catheters into region(s) of recurrent/progressive glioblastoma multiforme, anaplastic astrocytoma, or gliosarcoma defined by MRI. M032 is a second-generation oncolytic herpes simplex virus (oHSV) that is conditionally replication competent; replicating in tumor cells, but not in normal cells. The viral reproduction within transformed cells results in death, while simultaneously serving as a source of new vehicle to infect other tumor cells in the vicinity. Aside from the direct oncolytic activity, the virus carries a vector that promotes IL-12 expression, thereby reinforcing the local immune-mediated antitumor effect. An alternative viral-mediated immunotherapeutic approach is currently recruiting patients diagnosed with GBM for treatment with Ad-hCMV-TK and Ad-hCMV-Flt3L (NCT01811992). Similar to the preclinical studies, this therapeutic approach is hypothesized to be mediated by both a direct cytolytic and immune-component against glioma cells. Both trials offer promising hope for an alternative method of inducing productive tumor immunity with results that are highly anticipated.

Similar to other immunotherapies, gene therapy may work best if used as part of a combinatorial approach. Preclinical data suggest that gene therapy may be effective if it is combined with dendritic cell vaccination. In an experimental murine intracranial glioma model, combining IFN-β gene therapy with tumor cell lysate-pulsed DCs significantly prolonged survival compared to the monotherapies (15). This study highlights the importance of evaluating gene therapy approaches in their capacity to synergize with other immunotherapies.

**Supplementary Bibliography**

1. Weller M, Malipiero U, Aguzzi A, Reed JC, and Fontana A. Protooncogene bcl-2 gene transfer abrogates Fas/APO-1 antibody-mediated apoptosis of human malignant glioma cells and confers resistance to chemotherapeutic drugs and therapeutic irradiation. *The Journal of clinical investigation.* 1995;95(6):2633-43.

2. Jean WC, Spellman SR, Wallenfriedman MA, Hall WA, and Low WC. Interleukin-12-based immunotherapy against rat 9L glioma. *Neurosurgery.* 1998;42(4):850-6; discussion 6-7.

3. Yoshida Y, Sadata A, Zhang W, Saito K, Shinoura N, and Hamada H. Generation of fiber-mutant recombinant adenoviruses for gene therapy of malignant glioma. *Hum Gene Ther.* 1998;9(17):2503-15.

4. Parker JN, Gillespie GY, Love CE, Randall S, Whitley RJ, and Markert JM. Engineered herpes simplex virus expressing IL-12 in the treatment of experimental murine brain tumors. *Proceedings of the National Academy of Sciences of the United States of America.* 2000;97(5):2208-13.

5. Yamanaka R, Yajima N, Tsuchiya N, Honma J, Tanaka R, Ramsey J, Blaese M, and Xanthopoulos KG. Administration of interleukin-12 and -18 enhancing the antitumor immunity of genetically modified dendritic cells that had been pulsed with Semliki forest virus-mediated tumor complementary DNA. *J Neurosurg.* 2002;97(5):1184-90.

6. Chen B, Timiryasova TM, Haghighat P, Andres ML, Kajioka EH, Dutta-Roy R, Gridley DS, and Fodor I. Low-dose vaccinia virus-mediated cytokine gene therapy of glioma. *J Immunother.* 2001;24(1):46-57.

7. Merigan TC. Host defenses against viral disease. *N Engl J Med.* 1974;290(6):323-9.

8. Okada H, Lieberman FS, Edington HD, Witham TF, Wargo MJ, Cai Q, Elder EH, Whiteside TL, Schold SC, Jr., and Pollack IF. Autologous glioma cell vaccine admixed with interleukin-4 gene transfected fibroblasts in the treatment of recurrent glioblastoma: preliminary observations in a patient with a favorable response to therapy. *J Neurooncol.* 2003;64(1-2):13-20.

9. Friese MA, Platten M, Lutz SZ, Naumann U, Aulwurm S, Bischof F, Buhring HJ, Dichgans J, Rammensee HG, Steinle A, et al. MICA/NKG2D-mediated immunogene therapy of experimental gliomas. *Cancer research.* 2003;63(24):8996-9006.

10. Morioka J, Kajiwara K, Yoshikawa K, Ideguchi M, Uchida T, and Suzuki M. Vaccine therapy for murine glioma using tumor cells genetically modified to express B7.1. *Neurosurgery.* 2004;54(1):182-9; discussion 9-90.

11. Xu G, Jiang XD, Xu Y, Zhang J, Huang FH, Chen ZZ, Zhou DX, Shang JH, Zou YX, Cai YQ, et al. Adenoviral-mediated interleukin-18 expression in mesenchymal stem cells effectively suppresses the growth of glioma in rats. *Cell Biol Int.* 2009;33(4):466-74.

12. Curtin JF, Candolfi M, Fakhouri TM, Liu C, Alden A, Edwards M, Lowenstein PR, and Castro MG. Treg depletion inhibits efficacy of cancer immunotherapy: implications for clinical trials. *PLoS One.* 2008;3(4):e1983.

13. Curtin JF, Liu N, Candolfi M, Xiong W, Assi H, Yagiz K, Edwards MR, Michelsen KS, Kroeger KM, Liu C, et al. HMGB1 mediates endogenous TLR2 activation and brain tumor regression. *PLoS medicine.* 2009;6(1):e10.

14. Pluhar GE, Grogan PT, Seiler C, Goulart M, Santacruz KS, Carlson C, Chen W, Olin MR, Lowenstein PR, Castro MG, et al. Anti-tumor immune response correlates with neurological symptoms in a dog with spontaneous astrocytoma treated by gene and vaccine therapy. *Vaccine.* 2010;28(19):3371-8.

15. Saito R, Mizuno M, Nakahara N, Tsuno T, Kumabe T, Yoshimoto T, and Yoshida J. Vaccination with tumor cell lysate-pulsed dendritic cells augments the effect of IFN-beta gene therapy for malignant glioma in an experimental mouse intracranial glioma. *Int J Cancer.* 2004;111(5):777-82.
